# Prognostic Abilities and Quality Assessment of Models for the Prediction of 90-Day Mortality in Liver Transplant Waiting List Patients

**DOI:** 10.1371/journal.pone.0170499

**Published:** 2017-01-27

**Authors:** Ricardo Salinas Saldaña, Harald Schrem, Marc Barthold, Alexander Kaltenborn

**Affiliations:** 1 Core Facility Quality Management & Health Technology Assessment in Transplantation, Integrated Research and Treatment Center Transplantation (IFB-Tx), Hannover Medical School, Hannover, Germany; 2 Department of General, Visceral and Transplant Surgery, Hannover Medical School, Hannover, Germany; 3 Department of Trauma and Orthopedic Surgery, Federal Armed Forces Hospital Westerstede, Westerstede, Germany; Chiba University, Graduate School of Medicine, JAPAN

## Abstract

**Background:**

Model of end-stage liver disease (MELD)-score and diverse variants are widely used for prognosis on liver transplant waiting-lists.

**Methods:**

818 consecutive patients on the liver transplant waiting-list included to calculate the MELD, MESO Index, MELD-Na, UKELD, iMELD, refitMELD, refitMELD-Na, upMELD and PELD-scores. Prognostic abilities for 90-day mortality were investigated applying Receiver-operating-characteristic-curve analysis. Independent risk factors for 90-day mortality were identified with multivariable binary logistic regression modelling. Methodological quality of the underlying development studies was assessed with a systematic assessment tool.

**Results:**

74 patients (9%) died on the liver transplant waiting list within 90 days after listing. All but one scores, refitMELD-Na, had acceptable prognostic performance with areas under the ROC-curves (AUROCs)>0.700. The iMELD performed best (AUROC = 0.798). In pediatric cases, the PELD-score just failed to reach the acceptable threshold with an AUROC = 0.699. All scores reached a mean quality score of 72.3%. Highest quality scores could be achieved by the UKELD and PELD-scores. Studies specifically lack statistical validity and model evaluation.

**Conclusions:**

Inferior quality assessment of prognostic models does not necessarily imply inferior prognostic abilities. The iMELD might be a more reliable tool representing urgency of transplantation than the MELD-score. PELD-score is assumedly not accurate enough to allow graft allocation decision in pediatric liver transplantation.

## Introduction

The Model of End-Stage Liver Disease (MELD) Score has originally been developed as a prognostic model to estimate 90-day mortality for patients who require a transjugular intrahepatic portosystemic shunt procedure [1].The original MELD score is based on three laboratory values including serum creatinine, serum bilirubin and the International Normalized Ratio (INR) and the cause of cirrhosis [1]. In 2001, Kamath et al. evaluated the Model of End-Stage Liver Disease (MELD) Score as a prognostic model in patient groups with a broader range of disease severity and etiology and suggested its application in donor liver allocation policies for liver transplantation [2]. Subsequently, Wiesner et al. assessed the capability of the MELD Score without including the cause of cirrhosis into the MELD Score formula to correctly rank potential liver recipients according to their severity of liver disease and mortality risk on the OPTN liver waiting list in the US. They were able to show that the MELD score can accurately predict 90-day mortality among patients with chronic liver disease on the liver waiting list and can be applied for allocation of donor livers (see Table 1) [3]. Today, the MELD Score is applied in liver allocation policies in several countries world-wide, including the US and Germany [4, 5]. Since its introduction in German allocation policies, waiting list mortality has decreased from approximately 20% to 10% while post-transplant patient survival has declined significantly leading to 1-year survival rates that are up to 20% lower as compared to the United States and the United Kingdom [6–9]. It is astonishing that the MELD score has been introduced into liver allocation policies in Germany in December 2006 without prior validation of the prognostic ability of this prognostic model in German waiting list patients violating one of the essential quality assessment criteria for prognostic models as proposed by Jacob et al. in 2005 [10].

**Table 1:** Investigated prognostic models, their formulas and the handling of the required variables as published previously.

Score	Formula for calculation	Details
MELD (Wiesner et al., 2003)	10 x {0.957 x ln (creatinine[mg/dl]) + 0.378 x ln (bilirubin[mg/dl]) + 1.120 x ln (INR) + 0.643}	Score multiplied by 10 and rounded to the nearest integer. Maximum of 40 points. Laboratory values including INR, bilirubin and creatinine values < 1.0 are set to 1.0. Creatinine values above 4.0 mg/dl are set to 4.0 mg/dl. Same applies for patients who are under dialysis.
MESO Index (Huo et al., 2007)	[MELD/SNa (mmol/l)] x 10	MELD (as above) laboratory values < 1mg/dl are set to 1.0 mg/dl. Creatinine values above 4.0 mg/dl are set to 4.0 mg/dl. Same applies for patients who are under dialysis. SNa = serum Na^+^.
MELD Na (Kim et al., 2008)	MELD–Na^+^ (mmol/l)–[0.025 x (MELD) x (140-Na^+^ (mmol/l))] + 140	Na^+^ range of 125–140 mmol/l, lower values and larger values are rounded to the nearest integer in this range. Laboratory values < 1.0 are set to 1.0. Creatinine values above 4.0 mg/dl are set to 4.0 mg/dl. Same applies for patients who are under dialysis, rounded to the nearest integer.
UKELD (Barber et al., 2011)	(1.485 x ln (creatinine [μmol/l])) + (3.13 x ln (bilirubin [μmol/l])) + (5.395 x ln (INR))–(81.565 x ln (Na^+^ [mmol/l])) + 435	With a creatinine range of 1–400 μmol/l and Na^+^ range of 112–150 mmol/l. Values outside of these ranges are capped. Bilirubin values below 1.0 μmol/l are set to 1.0 μmol/l, INR values below 1.0 are set to 1.0.
iMELD(Luca et al., 2007)	MELD + [recipient age (years) x 0.3]—[0.7 x Na^+^(mmol/l)] + 100	MELD was used as described above.
refitMELD (Leise et al., 2011)	8.485 x ln (creatinine [mg/dl]) + 4.082 x ln (bilirubin [mg/dl]) + 10.671 x ln (INR) + 7.432	With a creatinine range of 0.8–3 mg/dl and INR range of 1.0–3.0. Values outside of these ranges are capped. Bilirubin values below 1.0 mg/dl are set to 1.0 mg/dl. For patients who are under dialysis, creatinine is set to 3 mg/dl.
refitMELD Na (Leise et al., 2011)	6.792 x ln (creatinine [mg/dl]) + 4.258 x ln (bilirubin [mg/dl]) + 8.29 x ln (INR) + 0.652 x (140—Na^+^ [mmol/l]) − 0.194 x (140—Na^+^ [mmol/l]) x (BiliCC [mg/dl]) + 6.327	With a Na^+^ range of 125–140 mmol/l. Values outside of these ranges are capped. Values of creatinine, bilirubin, and INR are defined as for the refitMELD. BiliCC is the same as bilirubin with values > 20 are set to 20. For patients who are under dialysis, creatinine is set to 3 mg/dl.
upMELD (Sharma et al., 2008)	1.266 x ln (1 + creatinine [mg/dl]) + 0.939 x ln (1 + bilirubin [mg/dl]) + 1.658 x ln (1 + INR)	Values of bilirubin, creatinine and INR < 1.0 are set to 1.0 mg/dl. Creatinine values > 4.0 mg/dl are set to 4.0 mg/dl with or without renal replacement therapy.
PELD (McDiarmid et al., 2002)	(0.436 x [if age <1 year = 1, otherwise = 0]* – 0.687 x ln (albumin [g/dl]) + 0.480 x ln (total bilirubin [mg/dl]) + 1.857 x ln [INR] + 0.667 x [growth failure: yes = 1, no = 0**]) x 10	* If the patient is less than one year old (scores for patients listed for liver transplantation before listed for liver transplantation before the patient´s first birthday continue to include the value assigned for age (<1 year) until the patient reached the age of 24 months) ** A patient has growth failure (<-2 standard deviation) if either the patient’s height is less than or equal to the expected sex- and age-matched low height value or the patient’s weight is less than or equal to the expected sex- and age-matched low weight value. The OPTN/UNOS PELD Calculator is used for candidates who are under 12 years old.

* If the patient is less than one year old

**Growth failure according to PELD was defined as summarized in http://www.unos.org/docs/MELD_PELD_Calculator_Documentation.pdf. Growth failure was set to 0 for all patients older than 219 months.

Since the introduction of MELD-Score based liver allocation in several countries, further prognostic models have been developed to predict 90-day mortality in liver transplant candidates including the MELD-sodium Index (MESO Index), MELD-Natrium-Score (MELD Na),United Kingdom End-Stage Liver Disease Score (UKELD),integrated MELD (iMELD), Revised model for End-Stage Liver Disease (refitMELD), revised model for End-Stage Liver Disease including sodium (refitMELDNa) and updated MELD Score (upMELD) (Table 1) [11–16]. For pediatric patients, the Pediatric End-Stage Liver Disease (PELD) score has been developed (Table 1) [17]. All of the above mentioned prognostic models have so far not been validated in German waiting list patients. The current study aims to validate the above mentioned prognostic models in a separate cohort of waiting list patients from Germany and to assess the fulfillment of the quality assessment criteria for prognostic models as proposed by Jacob et al. [10].

## Patients and Methods

### Inclusion and exclusion criteria

This is a single-center retrospective study including all waiting list patients (n = 818) listed for liver transplantation at Hannover Medical School between the 01.01.2007 and the 31.12.2013, who were either transplanted during that time interval or delisted due to clinical improvement or death (464 males (56.7%), 354 females (43.3%), median age at listing 46.0 years, range 0.02–73.6 years). Cases that were still on the waiting list for liver transplantation after the 31.12.2013 were excluded from analysis. Pediatric patients (n = 232, 28.4%) were defined as younger than 17 years due to specific donor organ allocation policies for these patients [5, 18]. The distribution of relevant clinical characteristics is summarized in Table 2.

**Table 2:** Clinical characteristics of the investigated cohort.

Variables	Distribution
Number of patients (total)	n = 818
Cohort description	
Age in years (median)	46 (0.02–73.63)
Male gender	n = 464 (56.7%)
Growth failure (age <16 years)*	n = 43 (18.5%)
Pediatric case (age < 16 years)	n = 232 (28.4%)
Pediatric case (age < 12 years)	n = 209 (25.6%)
High urgency status (ET)	n = 166 (20.3%)
Standard exception (ET)	n = 209 (25.6%)
Days on the waiting list (median)	91.5 (0–2323)
Weight in kg (median)	66 (2.4–140)
Height in cm (median)	1.68 (0.48–2.00)
Body mass index in kg/m^2^ (median)	22.8 (10.5–48.4)
Hemodialysis	n = 60 (7.3%)
Laboratory values	
Creatinine in μmol/l (median)	70 (0.43–1100)
Bilirubin in μmol/l (median)	66 (1.6–1710)
INR (median)	1.4 (0.9–21.2)
Sodium in mmol/l (median)	138 (121–161)
PTT in s (median)	41 (22–160)
Albumin in g/dl (median)	31 (3–53)
Indications	
Acute/subacute hepatic failure	n = 91 (11.1%)
Cholestatic liver disease	n = 74 (9.0%)
Congenital biliary disease	n = 93 (11.4%)
Liver cirrhosis	n = 234 (28.6%)
Cancers	n = 112 (13.7%)
Metabolic diseases	n = 70 (8.6%)
Budd Chiari syndrome	n = 10 (1.2%)
Benign or polycystic disease	n = 28 (3.4%)
Retransplantation case	n = 138 (16.9%)
Other liver diseases	n = 5 (0.6%)
MELD derivates and PELD	
MELD-score (median)	17.73 (6–40)
MESO Index (median)	1.28 (0.45–3.31)
MELD Na (median)	19.7 (6–40)
UKELD (median)	54.52 (39.9–76.7)
iMELD(median)	33.2 (-0.3–71.8)
refitMELD(median)	17.38 (5.5–43.5)
refitMELD Na (median)	12.93 (-25.56–37.71)
upMELD(median)	4.06 (2.68–8.73)
PELD in all age groups ** (median)	-9.06 (-36–39)
PELD in children <12 years (median)	-6.58 (-36–28)
PELD in children < 16 years(median)	-6.92 (-36–28)

The grouping of the indications leading to liver transplantation was performed according to the ELTR registry (http://www.eltr.org/).

*Growth failure according to PELD was defined as summarized in http://www.unos.org/docs/MELD_PELD_Calculator_Documentation.pdf.

** Growth failure was set to 0 for all patients older than 219 months. The percentage refers only to the pediatric sub-cohort age < 16 years. Continuous variables are given as median with range.

### Calculation of the investigated prognostic models and scores

Table 1 summarizes the equations and handling of variables for the calculation of the investigated scores including MELD, MESO Index, MELD-Na, UKELD, iMELD, refitMELD, refitMELD-Na, upMELD and PELD, as previously described [3, 11–17]. These scores have been analyzed as prognostic models for the prediction of study endpoints.

### Quality assessment of prognostic models

**Fig 1:**
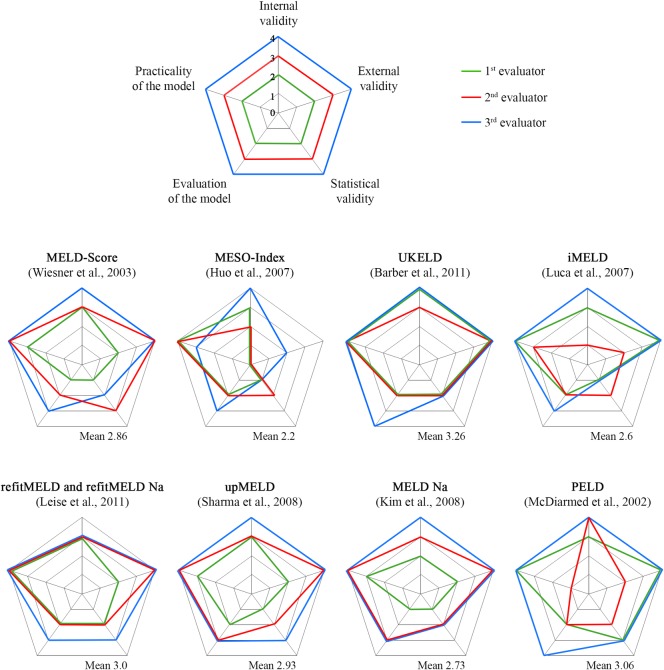
Quality Assessment of the nine investigated prognostic models as spider web illustration. Each corner of the pentagonal spider web represents one category of the quality assessment tool (internal validity, external validity, practicality of the model, evaluation of the model, and statistical validity). Each color represents one evaluator. An optimal assessment result would be an outer line connecting the five corner points. The nearer the line is at the center of the spider web, the poorer is the category’s assessed quality.

Quality assessment of the investigated prognostic models was carried out using the quality assessment tool for prognostic models in transplantation as proposed by Jacob and co-workers [10]. Assessment of study quality evaluated the criteria internal quality (quality subheadings 1–4), external validity (quality subheadings 1–2), statistical validity (quality subheadings 1–4), evaluation of the model (quality subheadings 1–4) and practicality of the model (quality subheadings 1–4) (see Table 3). Each investigated prognostic model was judged by the quality criteria described in detail under the respective quality subheadings by giving zero or one point for each subheading depending on the fulfilment of the respective quality criteria leading to an overall minimum of zero points and a maximum of 20 points per assessed study. To achieve an equal balance in the weighing between the individual quality subheadings, the score for the two categories of external validity was multiplied by the factor two. This assessment was made independently by three authors (R.S., A.K. and M.B.), all questions and doubts regarding the quality assessment of each subheading were documented and discussed. Furthermore spider web diagrams were created for each of the prognostic models (Fig 1).

**Table 3:** Shown is the quality assessment tool for prognostic models basing on Jacob et al. [10].

Tools for quality assessment of prognostic models	MELD	MESO-Index	UKELD	iMELD	refitMELD	refitMELDNa	upMELD	MELDNa	PELD
Internal validity									
**Inception cohort established**	0.67	1	1	1	1	1	1	1	1
**Inception cohort followed up**	1	1	1	1	0.67	0.67	1	1	1
**Baseline data collected prospectively**	1	0.33	0.67	0.33	0.67	0.67	0.67	0.67	0.67
**Candidate prognostic factors clearly defined**	0.67	0.67	0.67	0.67	0.67	0.67	0.67	0.67	1
External validity									
**Multi-centre population**	2	0	2	2	2	2	2	2	2
**Adequate description**	1.34	0.67	2	1.34	1.34	1.34	1.34	1.34	1.34
Statistical validity									
**Continuous variables**	0.67	0.67	0.67	0.67	1	1	0.67	0.67	1
**Sample size adequate**	1	0.67	1	0.67	1	1	1	1	1
**Collinearity between the candidate prognostic factors assessed**	0	0	0.33	0	0.33	0.33	0.33	0	0
**Missing values**	0.33	0	0	0	0	0	0	0	0.67
Evaluation of the model									
**Assumptions of the final model tested**	0.67	1	1	1	1	1	1	0.67	0.67
**Sensitivity of the final model to influential observations**	0.67	1	0.33	1	0	0	0.33	0.33	1
**Model validated with internal data**	0.33	1	0.33	1	0	0	0.33	0.33	1
**Model validated with external data**	0.33	0.33	1	0.67	1	1	1	1	0.67
Practicality of the model									
**Prognostic factorsavailable in clinical practice**	1	1	1	1	1	1	1	1	1
**Final model described sufficiently to fit to other data**	1	1	1	0.67	1	1	1	1	0.67
**Precision of the final model predictions**	0.67	1	1	1	1	1	0.67	0.67	0.67
**Potential to have wide generalisability**	1	0.67	1	1	1	1	1	1	0.67
**Total:**	14.32	11	16	14.02	15.01	15.01	15.01	14.35	15.36

Given are the mean values for quality assessment as observed in this study.

Each corner of the pentagonal spider web represents one category of the quality assessment tool (internal validity, external validity, practicality of the model, evaluation of the model, and statistical validity). Each color represents one evaluator. An optimal assessment result would be an outer line connecting the five corner points. The nearer the line is at the center of the spider web, the poorer is the category’s assessed quality.

### Study endpoints

The primary study endpoint was 90-day mortality on the transplant waiting list. The analyses strived to facilitate a comparison of the areas under the receiver operating characteristic (AUROC) curves for the prediction of 90-day mortality with the investigated prognostic models using data from the complete cohort as well as clinically relevant sub cohorts including pediatric and adult waiting list candidates, as well as waiting list candidates with specific indications for liver transplantation. Secondary study endpoint was the comparison of investigated prognostic models by using the mean results of the quality criteria assessment scores provided by three evaluators. Identification of independent risk factors for 90-day mortality with the goal to find possible explanations for differences in prognostic model performances in the investigated cohort was performed.

### Statistical analysis

This study was performed in accordance with the Transparent reporting of a multivariable prediction model for individual prognosis or diagnosis statement (TRIPOD) to guarantee highest possible quality standards [19].

Receiver Operating Characteristic (ROC)-curve analysis was performed to calculate the sensitivity, specificity, and overall model correctness of the investigated prognostic models. AUROCs larger than 0.700 indicate a potentially clinically useful prognostic model [20–23].

The relevance of variables as risk factors for the study endpoints was analyzed with binary logistic regression analysis. All statistically significant risk factors from univariable analyses have been taken into account for multivariable risk-adjusted models after exclusion of collinearity to identify independent risk factors for 90-day mortality (likelihood forward ratio inclusion method). For all statistical tests a p-value <0.05 was defined as significant. The SPSS statistics software version 21.0 (IBM, Somers, NY, USA) was used to perform statistical analysis.

### Ethical considerations

The institutional review board of the Hannover Medical School reviewed and approved this study (approval decision number 1683–2013). All patients have agreed that their data may be used for scientific purposes. All data were fully anonymized and de-identified by the primary investigator (last author) before it was accessed for this study.

Primary data cannot be published with the manuscript due to local institutional policy restrictions. However, fully anonymized and de-identified data will be made available upon request by the corresponding author.

None of the transplant donors were from a vulnerable population and all donors or next of kin provided written informed consent that was freely given.

## Results

### Events during follow-up

Mean follow-up was 228 days (median: 91.5 days, standard deviation (SD): 325.4 days, range: 0–2323 days) until liver transplantation (n = 567, 69.3%), patient’s death (n = 150, 18.3%) or delisting (n = 101, 12.3%). 90-day mortality on the waiting list was observed in 74 patients (9%). 31 of 101 delisted patients were delisted due to clinical improvement or stable disease, three patients were delisted due to progress of hepatocellular carcinoma, four patients due to incompliance, 17 patients due to transfer to another transplant center, 21 due to deterioration of their clinical condition, and three patients due to their own decision. For 22 patients no reasons for delisting were documented.

### Independent risk factors for 90-day-mortality

Univariable regression showed that age in years, creatinine in μmol/l, bilirubin in μmol/l, INR, sodium in mmol/l, PTT in s, albumin in g/dl, hemodialysis sessions per week (0–7), weight in kg, height in cm, acute/subacute hepatic failure, congenital biliary disease, body mass index in kg/m^2^, age < 16 years, and age < 12 years had a significant influence on 90-day mortality. Due to factor collinearity between body weight, body height and body mass index as well as between age and pediatric transplantation, only the more significant variables were included in multivariable modeling, which were BMI and age. To avoid collinearity between variables of the investigated scores and the scores themselves, two separate sets of risk-adjusted multivariable binary regression analyses were performed. The first risk-adjusted multivariable regression revealed creatinine (μmol/l), bilirubin (μmol/l), INR, sodium (mmol/l) and the body mass index (kg/m^2^) as independent risk factors for 90-day mortality (see regression analysis 1, Table 4).The second multivariable regression analysis included the MELD-score variants and revealed BMI, days on the transplant waiting list and the iMELD as independent risk factors for 90 day mortality.

**Table 4:** Results of univariable and multivariable regression analyses to determine the odds ratios of variables for the risk of 90-day waiting list mortality.

Variables	Univariable binary logistic regression			Risk-adjusted multivariable binary logistic regression model 1			Risk-adjusted multivariable binary logistic regression model 2		
	p-value	Odds Ratio	95% -CI	p-value	Odds Ratio	95% CI	p-value	Odds Ratio	95% CI
Age in years	<0.001	1.024	1.011–1.037				0.708	n.a.	n.a.
Male gender	0.457	n.a.	n.a.						
**Creatinine in μmol/l**	0.004	1.002	1.001–1.004	**0.031**	**1.002**	**1.000–1.004**			
**Bilirubin in μmol/l**	<0.001	1.002	1.001–1.003	**0.031**	**1.001**	**1.000–1.003**			
**INR**	<0.001	1.453	1.210–1.768	**0.006**	**1.459**	**1.116–1.907**			
**Sodium in mmol/l**	<0.001	0.908	0.867–0.951	**0.001**	**0.908**	**0.858–0.960**			
PTT in s	<0.001	1.022	1.014–1.031	0.176	n.a.	n.a.	0.218	n.a.	n.a.
Albumin in g/dl	0.007	0.949	0.913–0.986	0.545	n.a.	n.a.	0.700	n.a.	n.a.
Hemodialysis per week (0–7)	0.036	1.206	1.012–1.436	0.430	n.a.	n.a.	0.662	n.a.	n.a.
**Weight in kg**	<0.001	1.017	1.008–1.026						
Height in cm	0.007	3.165	1.377–7.273						
Acute/subacute hepatic failure	0.010	2.235	1.209–4.131	0.884	n.a.	n.a.	0.670	n.a.	n.a.
Cholestatic liver disease	0.579	n.a.	n.a.						
Congential biliary disease	0.021	0.097	0.013–0.707	0.174	n.a.	n.a.	0.919	n.a.	n.a.
Liver cirrhosis	0.065	n.a.	n.a.						
Cancers	0.267	n.a.	n.a.						
Metabolic diseases	0.467	n.a.	n.a.						
Budd Chiari syndrome	0.316	n.a.	n.a.						
Benign or polycystic disease	0.089	n.a.	n.a.						
Retransplantation case	0.867	n.a.	n.a.						
Other liver diseases	0.392	n.a.	n.a.						
**Body mass index in kg/m**^**2**^	0.001	1.065	1.025–1.107	**0.002**	**1.075**	**1.026–1.127**	**0.021**	**1.078**	**1.011–1.149**
Growth failure (age <16 years)*	0.170	n.a.	n.a.						
Pediatric case (age < 16 years)	0.001	0.281	0.133–0.596	0.142	n.a.	n.a.			
Pediatric case (age < 12 years)	0.002	0.280	0.127–0.621						
High urgency status (ET)	0.544	n.a.	n.a.						
Standard exception (ET)	0.031	0.482	0.249–0.934				0.816	n.a.	n.a.
Days on the waiting list	<0.001	0.988	0.983–0.993				**0.014**	**0.992**	**0.985–0.998**
MELD-score	<0.001	1.091	1.063–1.119				0.450	n.a.	n.a.
MESO Index	<0.001	3.549	2.483–5.074				0.680	n.a.	n.a.
MELD Na	<0.001	1.112	1.081–1.145				0.807	n.a.	n.a.
UKELD	<0.001	1.147	1.105–1.191				0.585	n.a.	n.a.
iMELD	<0.001	1.088	1.065–1.112				**0.001**	**1.060**	**1.024–1.098**
refitMELD	<0.001	1.102	1.072–1.133				0.730	n.a.	n.a.
refitMELD Na	0.510	n.a.	n.a.						
upMELD	<0.001	1.792	1.497–2.145				0.437	n.a.	n.a.
PELD in all age groups *	<0.001	1.055	1.033–1.078				0.574	n.a.	n.a.
PELD in children <12 years *	0.248	n.a.	n.a.						
PELD in children < 16 years *	0.213	n.a.	n.a.						

The grouping of the indications leading to liver transplantation was performed according to the ELTR registry (http://www.eltr.org/). Separate sets of multivariable regression were performed in order to avoid collinearity of related variables (n.a. = not applicable).

* Growth failure according to PELD was defined as summarized in http://www.unos.org/docs/MELD_PELD_Calculator_Documentation.pdf.

### Prognostic models as independent risk factors for survival

In the complete cohort the iMELD was the only independent risk factor for 90-day mortality in risk-adjusted multivariable binary logistic regression analysis of prognostic models (Table 4).

The PELD score could be identified as an independent risk factor for survival in all age groups in univariable analysis. It displayed a significant hazard for survival in both pediatric sub cohorts (age <12 years and age < 16 years).

### ROC-curve analysis results

The prognostic performance of the MELD variants and the PELD as prognostic models to predict 90-day mortality in the complete cohort and selected sub-cohorts is summarized in Table 5. For all patients the iMELD and the MELD Na, for children the MESO-Index and MELD Na and for adults the iMELD and the MELD Na displayed the largest areas under the ROC-curve (AUROCs) for this prediction (Table 5).

**Table 5:** Results of receiver operating curve (ROC) analysis with the determination of the area under the ROC-curve (AUROC) for the prediction of 90-day waiting list mortality.

	Area under the ROC-curve (95% confidence interval)								
	MELD	MESO- Index	UKELD	iMELD	refitMELD	refitMELD Na	upMELD	MELD Na	PELD
**All cases, n = 818**	0.736 (0.682–0.790)	0.751 (0.698–0.805)	0.752 (0.692–0.813)	**0.798 (0.749–0.847)**	0.750 (0.697–0.803)	0.588 (0.502–0.674)	0.719 (0.662–0.776)	**0.769 (0.717–0.821)**	0.692 (0.629–0.756)
**Children, n = 232**	0.745 (0.545–0.945)	**0.781 (0.553–1.000)**	0.702 (0.445–0.959)	0.724 (0.426–1.000)	0.737 (0.534–0.941)	0.519 (0.230–0.808)	0.712 (0.496–0.928)	**0.762 (0.516–1.000)**	0.699 (0.608–0.791)
**Adults, n = 586**	0.719 (0.662–0.776)	0.731 (0.675–0.788)	0.744 (0.682–0.806)	**0.760 (0.707–0.814)**	0.736 (0.680–0.791)	0.578 (0.488–0.667)	0.721 (0.663–0.779)	**0.752 (0.698–0.806)**	0.701 (0.633–0.770)
**No previous Tx, n = 680**	0.734 (0.677–0.791)	0.748 (0.691–0.804)	0.753 (0.689–0.816)	**0.801 (0.751–0.851)**	0.748 (0.693–0.803)	0.567 (0.473–0.662)	0.716 (0.656–0.775)	**0.769 (0.715–0.823)**	0.680 (0.610–0.750)
**Listed for Re-Tx, n = 138**	0.758 (0.604–0.913)	**0.792 (0.632–0.951)**	0.748 (0.560–0.936)	0.776 (0.610–0.941)	0.766 (0.608–0.924)	0.724 (0.535–0.913)	0.753 (0.584–0.922)	0.775 (0.601–0.949)	**0.785 (0.653–0.916)**
**No cirrhosis, n = 584**	0.775 (0.708–0.841)	0.796 (0.728–0.864)	0.795 (0.717–0.872)	**0.833 (0.767–0.900)**	0.785 (0.718–0.852)	0.550 (0.431–0.668)	0.763 (0.692–0.834)	**0.807 (0.740–0.875)**	0.749 (0.670–0.828)
**Cirrhosis, n = 234**	0.643 (0.548–0.739)	0.652 (0.558–0.747)	0.641 (0.541–0.742)	**0.683 (0.576–0.770)**	0.664 (0.572–0.756)	0.620 (0.500–0.740)	0.636 (0.539–0.733)	**0.674 (0.584–0.764)**	0.581 (0.471–0.691)
**No cancer, n = 706**	0.738 (0.681–0.794)	0.754 (0.698–0.810)	0.750 (0.686–0.814)	**0.797 (0.747–0.848)**	0.752 (0.697–0.807)	0.586 (0.495–0.677)	0.719 (0.658–0.780)	**0.773 (0.719–0.826)**	0.681 (0.611–0.750)
**Cancer, n = 112**	0.775 (0.600–0.949)	**0.782 (0.597–0.967)**	0.759 (0.553–0.965)	0.769 (0.556–0.981)	0.768 (0.560–0.976)	0.651 (0.384–0.918)	0.758 (0.576–0.940)	**0.786 (0.591–0.980)**	0.778 (0.561–0.994)
**No metabolic diseases, n = 748**	0.719 (0.661–0.778)	0.733 (0.674–0.791)	0.737 (0.672–0.801)	**0.785 (0.731–0.838)**	0.734 (0.677–0.791)	0.605 (0.516–0.693)	0.699 (0.638–0.760)	**0.752 (0.685–0.809)**	0.673 (0.605–0.742)
**Metabolic Diseases, n = 70**	0.855 (0.749–0.960)	0.891 (0.795–0.987)	0.873 (0.725–1.000)	**0.911 (0.818–1.000)**	0.865 (0.758–0.972)	0.458 (0.157–0.759)	0.873 (0.766–0.980)	**0.893 (0.805–0.981)**	0.818 (0.689–0.947)
**No acute/subacute hepatic failure, n = 727**	0.745 (0.688–0.802)	0.762 (0.704–0.819)	0.774 (0.711–0.837)	**0.823 (0.779–0.867)**	0.763 (0.708–0.819)	0.578 (0.480–0.676)	0.724 (0.664–0.785)	**0.785 (0.730–0.839)**	0.695 (0.628–0.762)
**Acute/subacute hepatic failure, n = 91**	0.650 (0.480–0.821)	0.642 (0.470–0.813)	0.607 (0.412–0.802)	**0.649 (0.476–0.823)**	0.640 (0.475–0.805)	0.538 (0.350–0.727)	0.636 (0.462–0.811)	0.648 (0.472–0.823)	**0.668 (0.460–0.877)**
**No cholestatic disease, n = 744**	0.730 (0.671–0.789)	0.744 (0.685–0.802)	0.734 (0.669–0.799)	**0.785 (0.732–0.839)**	0.746 (0.689–0.804)	0.628 (0.544–0.713)	0.715 (0.654–0.777)	**0.757 (0.700–0.815)**	0.695 (0.628–0.761)
**Cholestatic disease, n = 74**	0.795 (0.669–0.922)	0.818 (0.695–0.941)	**0.905 (0.829–0.980)**	**0.892 (0.817–0.967)**	0.788 (0.661–0.915)	0.195 (0.000–0.441)	0.750 (0.613–0.887)	0.861 (0.776–0.947)	0.669 (0.443–0.895)

The PELD displayed a good prognostic performance in patients listed for re-transplantation and for patients listed for acute/subacute hepatic failure in adults and children. The UKELD and the iMELD showed the strongest prognostic performance for waiting list patients with cholestatic diseases. For every other sub-cohort the iMELD and MELD Na displayed the best prognostic performance.

The refitMELD Na failed in all investigated sub-cohorts to predict outcome as measured by its AUROC. For cases with cirrhosis and acute/subacute hepatic failure all prognostic models displayed AUROCs <0.700 (Table 5).

### Quality assessment of prognostic models

The average quality assessment score of all investigated prognostic models was 14.45 points (72.25% of a maximum of 20 points), deploying the tool as suggested by Jacob et al. [10].

The MESO-Index (mean 11 score points) and the iMELD (mean 14.02 score points) reached the lowest overall quality scores based on their publications. The UKELD (mean 16 score points) and PELD (mean 15.36 score points) were rated with the highest overall quality scores (Table 3).

The lowest quality assessment scores were awarded for the quality subheadings statistical validity (mean: 0.49) and evaluation of the model (mean: 0.67).

Fig 1 shows the results of quality assessments of the three evaluators for the nine investigated prognostic models as spider web illustrations. Each corner of the pentagonal spider web represents one category of the quality assessment tool (internal validity, external validity, practicality of the model, evaluation of the model and statistical validity). An optimal assessment result would be an outer line connecting the five corner points. The nearer the line is at the center of the spider web, the poorer is the category´s assessed quality.

## Discussion

This is the first systematic evaluation of the quality and external validity of several prognostic models for 90-day mortality on the waiting list for liver transplantation. The quality of these models was assessed by three different investigators using the quality assessment tool proposed by Jacob et al. [10].The prognostic abilities of these models were assessed with an independent large data set from a single institution.

### Prognostic abilities of the investigated models

In many countries the investigated models have either already gained unsurpassed clinical importance for the allocation of donor livers for transplantation or have the potential for such use. This study shows that the iMELD clearly delivered a very high potential to indicate urgency of transplantation. In risk-adjusted multivariable binary logistic regression it was observed that the iMELD was the only score which could be revealed as significant independent risk factor for 90-day-mortality on the transplant waiting list. The already established prognostic scores like MELD and UKELD do not reach significance in risk-adjusted multivariable binary logistic regression in the current cohort.

Similar to other studies it can be confirmed that the MELD-score shows a good performance in ROC-curve analysis in all tested entities[24–27].Hence, the MELD-Score is an adequate tool to predict mortality on the transplant waiting list and therefore can be recommended as an integral part of liver allocation. However, the presented data suggests that there might be other prognostic models with a better performance regarding this highly relevant question, e.g. the iMELD and MELD Na. Both models show the best performance in most of the investigated sub-cohorts.

Interestingly, the refitMELD Na did not reach a larger AUROC than the previously developed refitMELD, although the refitMELD Na was published as an improved version of the latter score. This could also be shown in the investigated subgroups.

In the Eurotransplant community allocation of pediatric liver grafts is currently based on the recipients´ PELD-score. Therefore, it is somehow surprising that in this analysis the PELD score was not able to reach relevant AUROCs larger than 0.700 in the pediatric sub-cohort and is not able to reliably predict 90-day-mortality of children on the transplant waiting list. In this specific sub-cohort, the best AUROC was reached by the MESO-Index (0.781). These astonishing findings need to be further evaluated and a subsequent alteration of allocation mechanisms might be necessary to overcome this discrepancy.

This is further the case for the currently applied MELD-based allocation in adult liver transplantation since the MELD score reached an AUROC of 0.736, whereas the iMELD reached an AUROC 0.798. Thus, it is also recommended to investigate this issue systematically in a larger dataset.

### Quality assessment of the prognostic models

The systematic quality assessment of the underlying publications of the investigated prognostic models pointed out that there might be relevant quality issues in these studies. None of the models achieved maximum points in the quality assessment tool by three different investigators. Especially the statistical validity and the evaluation of the model showed room for improvement in most of the studies. It is obvious that many prognostic models were developed with no complete regard to the quality assessment criteria for prognostic models as proposed by Jacob et al. [10]. This may be due to a lack of an international consensus on the methodology that should be applied for the development of prognostic models, which was most recently suggested by the TRIPOD working party [19].

### Relation between quality assessment and prognostic abilities

Nevertheless, the current study shows that inferior quality assessment of prognostic models does not necessarily imply inferior prognostic value in this study´s cohort (e.g. iMELD). The reason for the differences between performance and quality could be the shortening of elementary information during the publication process. More transparence of the study design gives more confidence in the results and levels up the publication.

### Limitations of this study

During interpretation of this study´s results it should be taken into account that the underlying data is captured from a single center retrospective database, thus, there might be a possible center bias. This is further reflected by a comparatively small sample size. Therefore, further studies are needed to confirm the presented promising results, preferably with larger cohorts e.g. from transplant registries.

### Ethical requirements

The prioritization of patients and their timely access to an organ for liver transplantation frequently amounts to a decision on live and death. We therefore believe that very high ethical standards and a thorough evaluation of the quality and validity of prognostic models that are deployed for such use is mandatory. Such an evaluation includes an assessment of the internal, external and statistical validity, as well as the evaluation of model fit and clinical practicability. Jacob and colleagues proposed an excellent methodological approach as early as 2005 [10]. The recently published TRIPOD statement is an important step forward to qualitative prognostic research in transplantation and beyond [19].

A debate on the choice of the prognostic model that is intended to be applied for liver allocation and prioritization of liver transplantation should be guided by sound scientific data and a thorough quality assessment of the respective model. This would require a demonstration of the sensitivity, specificity and overall correctness of prediction of such a model including its model fit when applied on the population where it is intended to be deployed. Unfortunately, the MELD-score based liver allocation rules in Germany have been adopted without prior statistical evaluation in German liver transplant waiting list patients. The presented data suggest that the iMELD-score and the MELD-Na might provide a more accurate prediction of 90-day mortality on the transplant waiting list as compared to the MELD-Score, although this must be confirmed in larger studies.
